# Altered myocardial characteristics of the preexcited segment in Wolff-Parkinson-White syndrome: A pilot study with cardiac magnetic resonance imaging

**DOI:** 10.1371/journal.pone.0198218

**Published:** 2018-06-01

**Authors:** Hye-Jeong Lee, Jae-Sun Uhm, Yoo Jin Hong, Jin Hur, Byoung Wook Choi, Boyoung Joung, Young Jin Kim

**Affiliations:** 1 Department of Radiology, Research Institute of Radiological Science, Severance Hospital, Yonsei University College of Medicine, Seoul, South Korea; 2 Division of Cardiology, Cardiovascular Hospital, Severance Hospital, Yonsei University College of Medicine, Seoul, South Korea; Kurume University School of Medicine, JAPAN

## Abstract

**Purpose:**

The preexcited myocardium of Wolff-Parkinson-White (WPW) syndrome would have different characteristics from normal myocardium and these findings might be related to persistent left ventricular systolic dysfunction. We evaluated myocardial tissue characteristics at the preexcited segment in adult WPW syndrome patients and their implicated findings.

**Methods:**

For this prospective study, we enrolled 22 adult WPW syndrome patients (16 male, mean 45.4 ± 17.8 years) with echocardiographic findings of regional wall motion abnormality in our electrophysiology clinic. Of these patients, 14 underwent radiofrequency ablation before cardiac magnetic resonance imaging. All patients underwent cardiac magnetic resonance imaging including cine and late gadolinium enhancement. The ventricular morphology, function and myocardial characteristics of the preexcited segment were analyzed.

**Results:**

A relatively high prevalence of late gadolinium enhancement (9/22, 40.9%) was observed exclusively at the basal septum. The septal accessory pathway was significantly more prevalent in patients with late gadolinium enhancement (*P* = 0.011). The prevalences of regional myocardial wall thinning and regional akinesia were significantly higher (*P* = 0.001 for both) and left ventricular function was significantly decreased in patients with late gadolinium enhancement (*P* < 0.001). In addition, there were no significant relationships between radiofrequency ablation and regional akinesia (*P* > 0.999), regional myocardial wall thinning (*P* > 0.999), late gadolinium enhancement (*P* = 0.662) and low ejection fraction (*P* > 0.999).

**Conclusion:**

Myocardial fibrosis was observed at the preexcited myocardium of adult WPW syndrome patients with septal accessory pathway, which could accompany regional akinesia and regional myocardial wall thinning and might be related to persistent left ventricular systolic dysfunction even after radiofrequency ablation.

## Introduction

Wolff–Parkinson–White (WPW) syndrome is defined as a congenital condition involving an abnormal conductive accessory pathway between the atrium and ventricle that bypasses the atrioventricular node [1,2]. This syndrome is of clinical importance because it is frequently associated with supraventricular tachycardia. Furthermore, sudden cardiac death is potentially possible due to ventricular tachyarrhythmia from the associated atrial fibrillation with rapid anterograde conduction over the accessory pathway [3,4]. In addition, a rare cause of morbidity in WPW syndrome patients is heart failure, which may occur as a result of recurrent or sustained tachyarrhythmia [5]. Recently, several literatures have reported a possible direct association between WPW syndrome and heart failure, regardless of the related tachyarrhythmia [6–10]. Eccentric ventricular preexcitation through the accessory pathway results in premature contraction of that ventricle, and this has been well documented in echocardiography [11,12]. Regional premature contractions are thought to be a possible mechanism for heart failure in WPW syndrome by inducing progressive ventricular dilatation with cardiac dysfunction similar to functional aneurysm [6,13]. In addition, heart failure in WPW syndrome has been associated with the septal accessory pathway and has a reversible nature with a temporal relation after radiofrequency ablation (RFA) of the septal accessory pathway [7,8,10,14]. Cardiac magnetic resonance imaging (CMR) is a rapidly evolving technology that might now be the most powerful imaging tool for noninvasive myocardial characterization through the late gadolinium enhancement (LGE) and T1 mapping technique. Recent research has supported the value of these techniques for the assessment of myocardial characteristics under multiple conditions [15,16]. We thought that the preexcited segment in WPW syndrome could have different myocardial characteristics from the normal myocardium, especially in adult patients who are exposed to the accessory pathway for a long period of time, and we thought that the different characteristics could be related to left ventricular systolic dysfunction. However, as far as we know, CMR findings for myocardial characterization in WPW syndrome patients have rarely been studied. Therefore, we evaluated the myocardial characteristics of the preexcited segment using CMR in adult WPW syndrome patients and their implicated findings through this study.

## Materials and methods

### Study population

**Fig 1:**
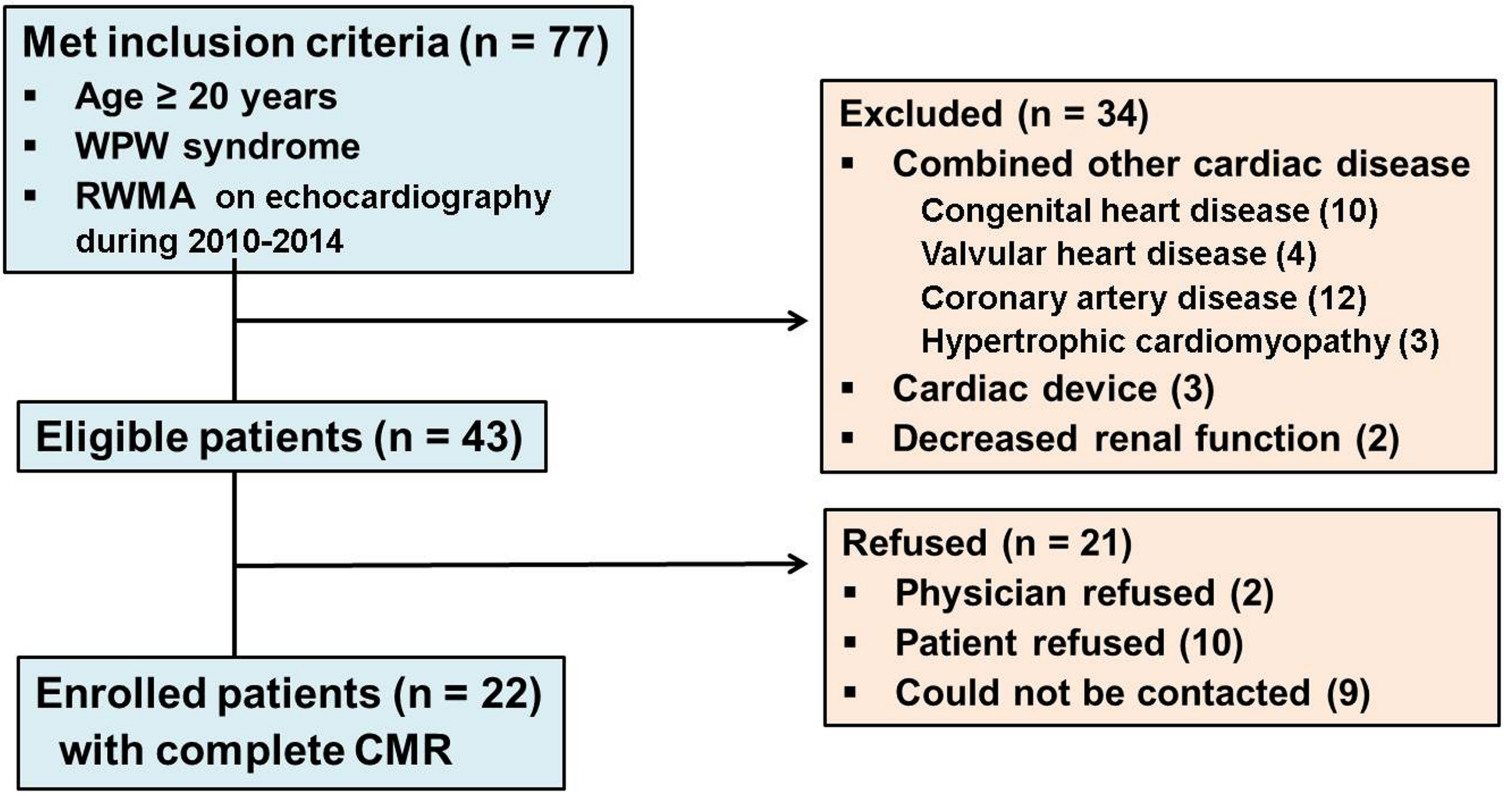
Flow chart for the study population. CMR = cardiac magnetic resonance imaging; RWMA = regional wall motion abnormality.

This prospective study was approved by our institutional review board and the local ethics committee. Written informed consent was obtained from all study participants. A cardiologist and a radiologist reviewed the electrical medical record database and searched for new adult WPW syndrome patients (age ≥ 20 years) who had undergone echocardiography at their first examination in our electrophysiology (EP) clinics between January 2010 and December 2014, and 327 patients were identified. Of them, 77 patients (23.5%, 77/327) who had abnormal echocardiographic findings of regional wall motion abnormality (RWMA), which did not correspond with vascular territory and was noted at the expected preexcited segment, were selected. A flow diagram for the study population and exclusion criteria is summarized in Fig 1. After the exclusion criteria were applied, 22 patients (16 males with a mean age of 45.4 ± 17.8 years) were finally enrolled. All 22 patients underwent CMR prospectively from January 2014 to May 2015.

### Clinical characteristics

All available clinical data were recorded after reviewing electronic medical records and/or after interviewing each patient with a standard questionnaire. Clinical presentation in terms of palpitation, chest pain and syncope was reviewed. The 12-lead electrocardiography (ECG) during sinus rhythm at the time of diagnosis was reviewed. On ECG, we reconfirmed ventricular preexcitation by Kent fibers with a short PR interval, widened QRS complex with slurred upstroke and secondary repolarization changes. An automatic computerized system recorded the longest QRS duration exhibited by the leads and the accessory pathway location was determined [17]. All study records from patients who underwent an EP study with RFA were reviewed for accessory pathways and related tachyarrhythmia. Data on performance of RFA and the approach method chosen for the ablation of the accessory pathway were also recorded. The accessory pathway locations were determined again in the EP study. From the results of the ECG and EP study, accessory pathway locations were categorized into one of the following three groups: septal (anteroseptal, midseptal and posteroseptal), right (right anterior, right lateral and right posterior), or left (left anterolateral, left lateral and left posterior) [18]. In addition, the presence of significant coronary artery disease was evaluated based on coronary artery computed tomography or conventional coronary angiography records obtained within 3 years before CMR.

### CMR protocol

CMR was performed with a 3.0-T imaging system (Magnetom Trio; Siemens Medical Solutions, Erlangen, Germany) and an 8-channel cardiac coil. After a localized scan, ECG-gated cardiac cine images were obtained with the following parameters: True fast imaging with steady-state free precession sequence, TR 3.31 ms, TE 1.44 ms, flip angle 50°, field of view 337 x 400 mm, matrix 216 x 216, 25 phases and slice thickness 8 mm without gap in the four-chamber, long-axis and short-axis planes encompassing the whole ventricles. LGE imaging was performed 10 minutes after injection of gadobutrol (0.2 mmol/kg, Gadovist; Bayer Schering Pharma AG, Berlin, Germany) at 2 ml/sec. Scanning parameters were as follows: segmented inversion recovery prepared turbo fast low-angle shot sequence, TR 9.9 ms, TE 4.9 ms, flip angle 20°, field of view 380 x 380 mm, matrix 320 x 320 and slice thickness 8 mm without gap in the four-chamber, long-axis and short-axis planes encompassing the whole ventricles. Data acquisition was synchronized with ECG in the mid-diastolic phase. An 11-heartbeat modified Look-Locker sequence with inversion recovery was used for T1 measurement of the myocardium [19]. Scanning parameters were as follows: TR/TE = 2.43/1.01 ms, minimum inversion time 100 msec with inversion time increment 80 ms, field of view 308 x 380 mm^2^, acquisition matrix 126 x 192, flip angle 35°, and slice thickness 8 mm. Short-axis images were acquired at the apical, mid and basal levels of the left ventricle. Images for T1 measurements were obtained before and 15 minutes after contrast administration.

### Image analysis

**Fig 2:**
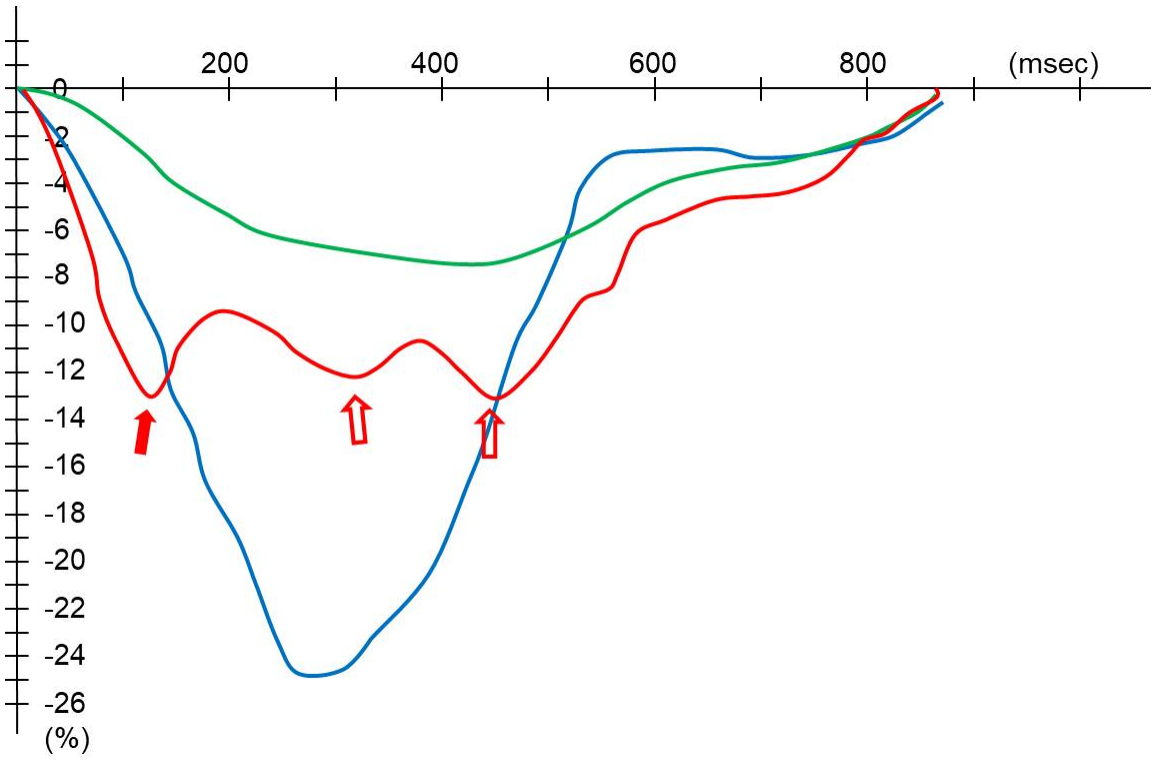
Strain curves for the regional wall motion abnormalities. Representative longitudinal strain curves are shown with the blue curve representing a normal contraction, the red a premature contraction, and the green hypokinesia or akinesia. Regional premature contraction was defined with the early systolic peak during the systole with decreased time to peak (red solid arrow) and overall decreased absolute values of global strains compared to the normal curve. An additional systolic peak (second or third) (red open arrows) could also indicate premature contraction. Regional hypokinesia or akinesia was defined with decreased absolute values of global strains and mild increased time to peak.

All CMR images were transferred to dedicated software (CMR 42; Circle Cardiovascular Imaging, Calgary, Alberta, Canada). Two observers (H.J.L and Y.J.K. with 9 and 12 years of experience in cardiac imaging, respectively) who were both blinded to each patient’s clinical findings reviewed the images in consensus. From the cine images, observers evaluated the presence of regional myocardial wall thinning, which was defined as a myocardial wall thickness of 5.5mm or less on the end-diastolic image [20]. To evaluate myocardial mechanics, a semi-automated feature tracking method was used to obtain information on the ventricular strains: radial, circumferential and longitudinal strains. Using the results for global strain, strain rate, and time to peak, observers determined the presence of RWMAs (Fig 2) [21]. Afterwards, both ventricular end-diastolic volumes with ejection fractions were calculated by semi-automatically tracing the endocardial contours at end-systole and end-diastole in each short-axis image from the apical to basal ventricle. Patients were regarded as having low ejection fraction if the left ventricular ejection fraction was < 50%. Myocardial LGE was defined as a region with an apparent high signal intensity of > 5 standard deviations of the remote normal myocardium. If LGE was detected, its pattern was further classified and the location was recorded. Myocardial extracellular volume fraction was measured using pre- and post-contrast T1 map images with the following equation: Extracellular volume fraction (%) = (ΔR1_m_/ΔR1_b_) x (1 –hematocrit) x 100, for which R1_m_ is R1 in the myocardium, R1_b_ is R1 in the blood, and ΔR1 is the change in relaxivity before and after gadolinium chelate administration, respectively [22].

### Statistical analysis

Statistical analyses were performed using statistical software (SPSS version 23.0 for Windows, SPSS). Continuous variables were expressed as mean values with standard deviations and categorical variables were expressed as percentages. We divided the patients into two groups according to the presence of LGE, and compared clinical characteristic and other CMR findings between the two groups. Additional analyses were done in which the study population was divided into two groups according to the performance of RFA or the presence of RWMA. The Mann-Whitney U test was used to appraise the differences between the two groups for continuous variables and the chi-square contingency tables or Fisher’s exact test were used to evaluate the differences between the two groups for categorical variables. A *P* value of < 0.05 was considered to indicate statistical significance.

## Results

### Clinical characteristics

The clinical characteristics of the study population are summarized in Table 1. The mean age of the patients was 43.6 ± 17.2 years at first diagnosis. All patients underwent ECG at the time of diagnosis and had ventricular preexcitation caused by Kent fibers on ECG. Palpitations were present in all patients. Of the patients with palpitations, chest pain was additionally documented in 6 patients and syncope in 4 patients. Fourteen patients of the study population underwent an EP study with RFA due to documented supraventricular tachyarrhythmias prior to CMR. The median time interval from first diagnosis to RFA for the 14 patients was 65.5 (IQR, 54.8–87.5) days. Accessory pathways were classified based on mainly ECG findings because not all patients underwent an EP study. For the 14 patients who underwent an EP study, both the ECG and EP study classified the accessory pathways identically. There was no patient with multiple accessory pathways. No recurrence was observed in patients with RFA. In addition, we observed no significant coronary artery disease in 16 patients on coronary computed tomographic angiography or conventional coronary angiography. The other 6 patients did not undergo coronary evaluation.

**Table 1:** Comparison of clinical characteristics according to the presence of LGE.

	All patients (n = 22)	LGE (-) (n = 13)	LGE (+) (n = 9)	*P* value
Male sex	16 (72.7%)	10 (76.9%)	6 (66.7%)	0.655
Age at diagnois (yrs)	43.6 ± 172	44.9 ± 19.5	41. ± 15.0	0.857
Palpitation	22 (100.0%)	13 (100.0%)	9 (100.0%)	0.376
Chest pain	6 (27.3%)	4 (30.8%)	2 (22.2%)	> 0.999
Syncope	4 (18.2%)	4 (30.8%)	0 (0.0%)	0.115
Age at MRI (yrs)	45.4 ± 17.8	46.9 ± 20.1	42.4 ± 15.4	0.756
QRS duration (msec)	141.3 ± 19.7	134.9 ± 18.6	150.4 ± 18.4	0.021
Tachyarrhythmia	14 (63.6%)	9 (69.2%)	5 (55.6%)	0.662
AVRT	10 (45.5%)	6 (46.2%)	4 (44.4%)	0.937
AF	4 (18.2%)	3 (23.1%)	1 (11.1%)	0.616
RFA	14 (63.6%)	9 (69.2%)	5 (55.6%)	0.662
Retrograde aortic	3 (13.6%)	1 (7.7%)	2 (22.2%)	0.544
Transseptal	11 (50.0%)	8 (61.5%)	3 (33.3%)	0.387
AP location				
Right	4 (18.2%)	3 (23.1%)	1 (11.1%)	0.616
Septal	14 (63.6%)	6 (46.2%)	8 (88.9%)	0.011
Left	4 (18.2%)	4 (30.8%)	0 (0.0%)	0.046

Values are mean ± standard deviation or patient number (%).

LGE = late gadolinium enhancement; AVRT = atrioventricular reentrant tachycardia; AF = atrial fibrillation; RFA = radiofrequency ablation; AP = accessory pathway

### CMR findings

Median time interval from first diagnosis to CMR was 499.5 (IQR, 352.8–754.0) days. Median time interval from RFA to CMR was 465.5 (IQR, 276.5–709.5) days for the 14 patients with RFA. The CMR findings from the study population are summarized in Table 2. On CMR, RWMA was noted at the preexcited segment in 13 patients (13/22, 59.1%) and all of these findings were observed at the basal septum. Regional premature contractions were noted in 6 patients with a septal accessory pathway and 1 patient with a right posterior accessory pathway close to the inferior septum. Regional akinesia instead of premature contraction was noted in 6 patients with a septal accessory pathway. On the cine images, regional myocardial wall thinning was noted at the basal septum in 6 patients with a septal accessory pathway (6/22, 27.3%). The mean values of the end-diastolic volume were 90.8 ± 30.9 ml/BSA and 78.8 ± 15.8 ml/BSA, and ejection fractions were 56.7 ± 12.0% and 58.0 ± 5.8% for the left and right ventricles, respectively. From the functional results, 7 patients (7/22, 31.8%) were classified as having low ejection fraction with a decreased left ventricular ejection fraction of < 50%. LGE at the preexcited segment was present in 8 patients with a septal accessory pathway and 1 patient with a right accessory pathway (9/22, 40.9%). All demonstrated LGEs were detected at the basal septum (both the anterior and inferior septum in 5 patients and only the inferior septum in 4 patients). The patterns of LGE were as follows: ill-defined patchy LGE in 4 patients and linear endocardial and/or epicardial LGE not corresponding with vascular territory in 5 patients. All of the 6 patients who did not undergo coronary evaluation did not show LGE on CMR. The native T1 value of the myocardium was measured as 1253.9 ± 41.6 msec and myocardial extracellular volume fraction was measured as 27.2 ± 1.8%.

**Table 2:** Comparison of CMR findings according to the presence of LGE.

	All patients (n = 22)	LGE (-) (n = 13)	LGE (+) (n = 9)	*P* value
Wall thinning	6 (27.3%)	0 (0.0%)	6 (66.7%)	0.001
RWMA	13 (5.1%)	4 (30.8%)	9 100.0%)	0.002
remature contraction	7 (31.8%)	4 (30.8%)	3 (33.3%)	> 0.999
Akinesia	6 (27.3%)	0 (0.0%)	6 (66.7%)	0.001
LVEDV (ml/BSA)	90.8 ± 30.9	76.0 ± 8.3	112.4 ± 38.9	0.003
LVEF (%)	56.7 ± 12.0	64.1 ± 5.0	45.9 ± 10.8	< 0.001
RVEDV (ml/BSA)	78.8 ± 15.8	76.0 ± 14.3	83.0 ± 17.8	0.601
RVEF (%)	58.0 ± 5.8	61.6 ± 2.6	52.7 ± 5.0	0.001
Low ejection fraction	7 (31.8%)	0 (0.0%)	7 (77.8%)	< 0.001
Native T1 (msec)*	1253.9 ± 41.6	1222.5 ± 11.6	1300.0 ± 20.5	< 0.001
Septal*	1287.0 ± 71.7	1230.7 ± 15.3	1368.4 ± 23.1	< 0.001
Lateral*	1220.8 ± 25.8	1214.2 ± 16.3	1230.2 ± 34.3	0.284
ECV (%)*	27.2 ± 1.8	26.1 ± 0.5	28.7 ± 1.8	0.006
Septal*	28.4 ± 3.4	26.0 ± 0.7	31.8 ± 2.8	< 0.001
Lateral*	26.3 ± 1.0	26.2 ± 0.6	26.5 ± 1.4	0.793

Values are mean ± standard deviation or patient number (%).

*Reference values of the normal volunteers (N = 7) on the same CMR system are presented as mean values with standard deviations as follows: Native T1 = 1245.4 ± 52.7 msec; 1249.6 ± 34.0 msec and 1253.5 ± 35.9 msec for septal and lateral, respectively. Myocardial ECV = 26.3 ± 1.4%; 26.3 ± 1.3% and 26.2 ±1.8% for septal and lateral, respectively.

LGE = late gadolinium enhancement; RWMA = regional wall motion abnormality; LVEDV = left ventricular end-diastolic volume; BSA = body surface area; LVEF = left ventricular ejection fraction; RVEDV = right ventricular end-diastolic volume; RVEF = right ventricular ejection fraction; ECV = extracellular volume fraction

### Differences in clinical and other CMR findings according to the presence of LGE

Among clinical findings, the QRS duration was significantly longer in patients with LGE compared to patients without LGE (*P* = 0.021). The prevalence of septal accessory pathway was significantly higher in patients with LGE compared to patients without LGE (*P* = 0.011). In addition, the prevalence of lateral accessory pathway was significantly lower in patients with LGE compared to patients without LGE (*P* = 0.046). Other findings did not show significant differences between the two groups. Among CMR findings, a significantly higher prevalence of regional myocardial wall thinning was observed in patients with LGE (*P* = 0.001). The prevalence of akinesia was also significantly different between the two groups (*P* = 0.001) with akinesia being more prevalent in patients with LGE. However, for premature contractions, there was no significant difference between the two groups (*P* > 0.999). The mean left ventricular end-diastolic volume was significantly larger (*P* = 0.003) and left ventricular ejection fraction was significantly decreased (*P* < 0.001) in patients with LGE. Likewise, the prevalence of low ejection fraction was significantly higher in patients with LGE (7/9, 77.8%) than patients without LGE (0/13, 0.0%) (*P* < 0.001). In addition, patients with LGE showed significantly decreased right ventricular ejection fraction compared to patients without LGE (*P* = 0.001). The mean values of myocardial native T1 and extracellular volume fraction were significantly higher in patients with LGE compared to patients without LGE (*P* < 0.001 and 0.006, respectively), especially at the septum (*P* < 0.001 for both), but not for the lateral wall of the left ventricle (*P* = 0.284 and 0.793, respectively).

### Comparison of major findings according to performance of RFA

The major findings were compared according to performance of RFA and the results are summarized in Table 3. Among the 14 patients who underwent RFA before CMR, 9 patients showed no RWMA but 5 patients still had RWMA on CMR even after successful RFA. The prevalence of regional premature contractions was significantly lower in patients who underwent RFA (*P* = 0.002). However, we observed no significant differences in the prevalences of regional akinesia and regional myocardial wall thinning between the two groups (*P* > 0.999 for both). In addition, there were no significant differences in the prevalences of LGE and low ejection fraction between the two groups (*P* = 0.662 and > 0.999, respectively).

**Table 3:** Comparison of major findings according to performance of RFA.

	RFA (+) (n = 14)	RFA (-) (n = 8)	*P* value
RWMA	5 (35.7%)	8 (100.0%)	0.006
Premature contraction	1 (7.1%)	6 (75.0%)	0.002
Akinesia	4 (28.6%)	2 (25.0%)	> 0.999
Wall thinning	4 (28.6%)	2 (25.0%)	> 0.999
LGE	5 (35.7%)	4 (50.0%)	0.662
Low ejection fraction	4 (28.6%)	3 (37.5%)	> 0.999

Values are patient number (%).

RFA = radiofrequency ablation; RWMA = regional wall motion abnormality; LGE = late gadolinium enhancement.

### Relationships between major findings with a set diagram

**Fig 3:**
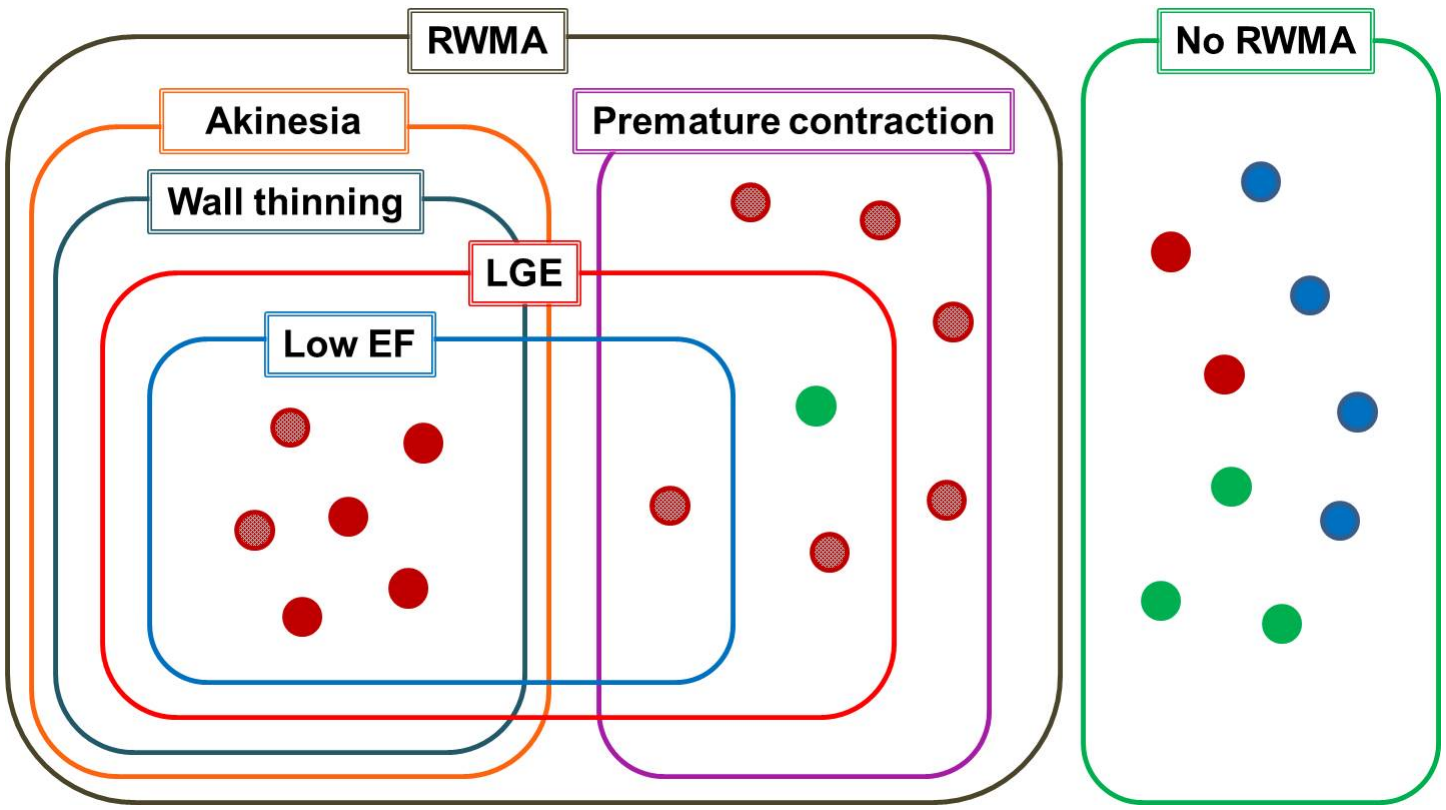
Set diagram for the logical relations between the major findings. Red circles indicate patients with septal accessory pathways, green circles indicate right accessory pathways and blue circles indicate left accessory pathways. Patterns within the circles indicate patients who did not undergo radiofrequency ablation. EF = ejection fraction; LGE = late gadolinium enhancement; RWMA = regional wall motion abnormality.

**Fig 4:**
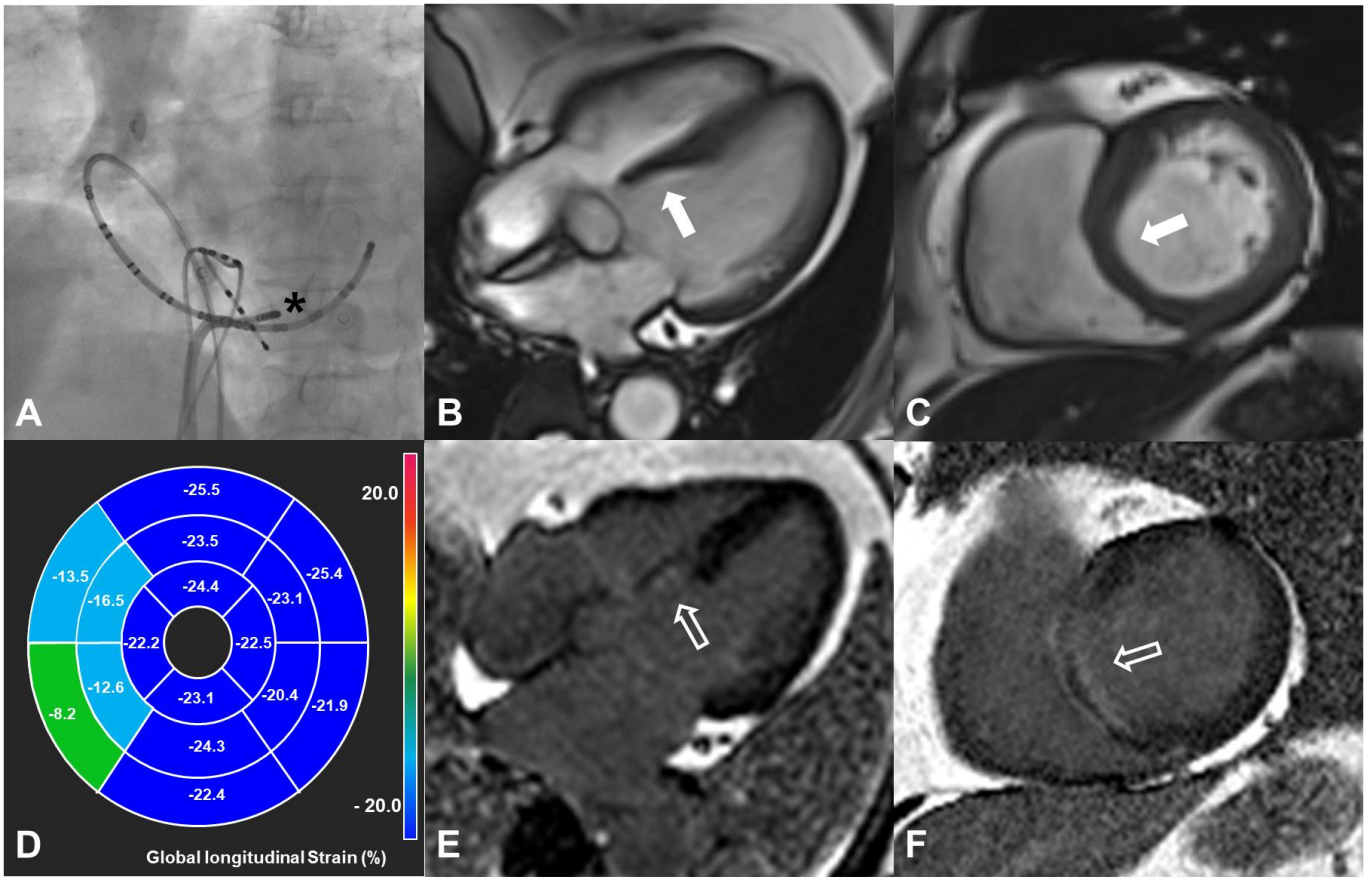
A 67-year-old female patient with Wolff-Parkinson-White syndrome. She underwent an electrophysiologic study due to frequent palpitations. Radiofrequency ablation was applied to the posteroseptal accessory pathway through the transseptal approach (asterisk) (A). One year later, the patient underwent cardiac magnetic resonance imaging. Four-chamber and short-axis cine images show regional myocardial wall thinning (about 5.1mm) at the basal inferior septum on the end-diastolic phase (arrows) (B, C). Decreased global longitudinal strain is noted at the basal septum with the feature tracking method, which suggests regional akinesia (D). The left ventricular ejection fraction is 47.6%. Linear endocardial and epicardial late gadolinium enhancements not corresponding with the vascular territory are noted on the four-chamber and short-axis late gadolinium enhancement images (open arrows) (E, F).

A set diagram was drawn to describe logical relations between the major findings (Fig 3). All 9 patients without RWMA received RFA before CMR and did not show low ejection fraction as well as LGE. Among the 13 patients with RWMA at the preexcited segment, 7 patients showed low ejection fraction and all these patients had LGE. Of the 7 patients with low ejection fraction, 1 patient had regional premature contractions and 6 patients had regional akinesia with myocardial wall thinning (Fig 4). Of the 6 patients without low ejection fraction from regional premature contractions, 2 patients showed LGE and 4 patients did not. All patients with regional akinesia had a septal accessory pathway; 4 patients underwent RFA and 2 patients did not. In addition, we observed that the prevalence of the septal accessory pathway (92.3%,12/13) was significantly higher in patients with RWMA compared to patients without RWMA (22.2%, 2/9) (*P* < 0.001) from the diagram. The prevalence of LGE in patients with RWMA (69.2%, 9/13) was also significantly higher than in patients without RWMA (0.0%, 0/9) (P < 0.001).

## Discussion

We evaluated myocardial characteristics of the preexcited myocardium using CMR in adult patients with WPW syndrome. In our results, we observed a relatively high prevalence of LGE in the study participants (9/22, 40.9%) exclusively at the basal septum. The prevalence of septal accessory pathway was significantly higher in patients with LGE. In addition, the prevalences of regional myocardial wall thinning and regional akinesia were significantly higher in patients with LGE. Left ventricular end-diastolic volume was significantly larger and both ventricular ejection fractions were significantly decreased in patients with LGE. The native T1 value and extracellular volume fraction were significantly increased in patients with LGE, especially at the septum, which might support the presence of myocardial fibrosis. In addition, there were no significant relationships between RFA and abnormal CMR findings such as regional akinesia, regional myocardial wall thinning, LGE and low ejection fraction in the present study.

Although we included patients with RWMA observed on echocardiography, only 13 patients (13/22, 59.1%) had RWMA on CMR. This is probably because the patients without RWMA on CMR underwent RFA before undergoing CMR. As ventricular preexcitation in the manifest WPW syndrome occurs at the myocardium close to the ventricular insertion of the accessory pathway, we thought that abnormal findings in the myocardium on CMR might be exclusively observed at the basal left ventricle [23]. In addition, all these abnormal findings were observed at the preexcited segment in patients with a septal accessory pathway, namely the basal septum, with the exception of 1 patient with a right posterior accessory pathway who had a preexcited segment close to the inferior septum. Hypothetically, this finding might be related to the relatively shorter conduction time between the sinus node and septal accessory pathway [7,8,13], which might have more effects on the myocardium compared to the right or left accessory pathways.

Recently published literatures have reported that non-physiologic activation through the accessory pathway can directly lead to heart failure [6–8,13,14,24]. According to this hypothesis, abnormally decreased local preload by premature contractions can decrease the workload of the preexcited myocardium, which might induce regional myocardial wall thinning [25–27]. Regional myocardial wall thinning could then induce the systolic bulging of the corresponding segment, which in turn progresses to heart failure. Likewise, these findings were exclusively observed at the basal septum in patients with a septal accessory pathway. Previous research has also reported that regional myocardial wall thinning and/or heart failure in WPW syndrome are reversible and that cardiac function can be restored after RFA. However, in the present study, we observed 9 patients (9/22, 40.9%) with LGE at the basal septum, which could suggest myocardial fibrosis. Also, regional myocardial wall thinning, regional akinesia and low ejection fraction were only present in patients with LGE. In addition, there were no significant differences between these abnormal findings and the performance of RFA. In the set diagram, we observed that 3 of the 7 patients with regional premature contractions showed LGE, with 1 patient showing low ejection fraction while the other 2 patients did not. The 4 patients with regional premature contractions without LGE did not present with low ejection fraction. From these findings, we deduced that continuous premature contractions might cause regional myocardial wall thinning, which might proceed to myocardial fibrosis and lead to gradual worsening of the RWMA from premature contractions to akinesia along with the occurrence of persistent left ventricular systolic dysfunction even after successful RFA.

The hitherto published literatures have almost all focused on echocardiographic findings in pediatric patients, whereas we focused on CMR for adult patients, who might be diagnosed later in life and who might be exposed to the accessory pathway for a long period of time. Hence, we observed different findings such as myocardial fibrosis that could be related to persistent left ventricular systolic dysfunction even after successful RFA. We thought that our research could also help explain why three pediatric patients in a previous study had irreversible heart failure in spite of RFA [6]. Expectably, we observed that premature contractions were not noted in patients with RFA except for 1 patient with right posterior accessory pathway, who had a short time interval between RFA and CMR. Prior studies have suggested that premature contractions of the ventricle can persist after successful RFA probably due to the existence of a cardiac memory, and that these abnormal contractions might gradually return to normal contractions over a 1-month period [28,29]. However, from the results of the present study, we assumed that a few patients could have myocardial fibrosis before RFA, which might progress to regional myocardial wall thinning and akinesia, and ultimately to persistent left ventricular systolic dysfunction in spite of RFA. From a different perspective, performing RFA would be a critical determinant of myocardial fibrosis. However, there was no significant difference in LGE according to RFA in the present study. In addition, RFA is usually applied to a very small area near the atrioventricular annulus after accessory pathway localization, but the areas of LGE were much larger than what would be expected with just ablation. Further prospective studies using CMR with a large study population might be necessary to confirm these findings.

This study has some limitations. First, we included a small number of patients in this preliminary study. Second, we selected eligible patients with grossly visible RWMA on echocardiography because non-visible RWMA can be related to very small areas of preexcited segment, and it would be difficult to characterize myocardium on CMR considering spatial resolution. In actual practice, however, not all patients with WPW syndrome would undergo echocardiography. This ultimately raises the possibility of a selection bias, and consequentially the incidence of LGE might have been higher than its actual value. Third, although we prospectively performed CMR in this study, not all patients underwent the EP study and clinical follow-up varied among the patients. To investigate clinical findings related to myocardial fibrosis and heart failure in patients with WPW syndrome, further prospective studies with larger populations are necessary.

## Conclusions

We observed a relatively high prevalence of myocardial fibrosis at the preexcited myocardium of adult WPW syndrome patients, exclusively at the basal septum with septal accessory pathway in the present study. In some cases, myocardial fibrosis accompanied regional akinesia and regional myocardial wall thinning. These abnormal findings might have occurred from continuous premature contractions by the septal accessory pathway and might be related to persistent left ventricular systolic dysfunction even after successful RFA. A further prospective study using CMR with a large population is necessary to identify myocardial characteristics at the preexcited myocardium in WPW syndrome, which might provide new perspectives for the disease and help develop management strategies in the future.
